# The Associations Between Obesity and Deep Vein Thrombosis in Patients With Cardiovascular Disease: A Narrative Review

**DOI:** 10.7759/cureus.66731

**Published:** 2024-08-12

**Authors:** Cynthia Sanchez, Katherine Miller, Rhea Raj, Kesava Mandalaneni, Sudhakar Pemminati, Vasavi R Gorantla

**Affiliations:** 1 School of Medicine, St. George's University, St. George, GRD; 2 School of Medicine, St. George's University, St. George's, GRD; 3 Neuroscience, Oakland University William Beaumont School of Medicine, Rochester Hills, USA; 4 Pharmacology, California Health Sciences University College of Osteopathic Medicine, Clovis, USA; 5 Biomedical Sciences, West Virginia School of Osteopathic Medicine, Lewisburg, USA

**Keywords:** therapeutic anticoagulation, pulmonary embolism, cardiovascular disease, obesity, deep vein thrombosis

## Abstract

This article provides an in-depth review of the relationship between obesity and deep vein thrombosis (DVT) in patients with cardiovascular disease (CVD). Our aim is to enhance understanding of the associations between obesity and DVT, particularly in patients with comorbid cardiovascular conditions. This relationship, although significant, is often underrepresented in discussions about obesity and DVT. Current research frequently lacks clarity on whether studies of obesity and DVT account for the presence of coexisting CVD.

We draw on data from systematic reviews, meta-analyses, and other peer-reviewed medical journals that focus on individuals who are overweight or obese and their association with DVT and CVD. The review begins with an introduction to cardiovascular disease, venous thromboembolic disease, and obesity. We then examine potential links between obesity and DVT, emphasizing the roles of gender, venous stasis, chronic inflammation, and decreased fibrinolytic activity.

Key findings suggest that while obesity may contribute to the development of DVT, this association is not significantly affected by adjustments for cardiovascular risk factors. The review highlights the need for further research, specifically targeting studies that investigate cardiovascular disease as an underlying risk factor in obese individuals who develop DVT.

## Introduction and background

Cardiovascular disease

Noncommunicable diseases, such as cardiovascular and respiratory diseases, cancers, and diabetes, are responsible for 74% of all deaths worldwide [1]. Cardiovascular disease (CVD) accounts for more than half of these noncommunicable diseases, corresponding to approximately 17.9 million people per year [1]. In the United States alone, more than 800,000 people die of CVD each year, equating to one in three deaths annually [2]. According to the Centers for Disease Control and Prevention (CDC) chapter on Heart Disease, CVD is the leading cause of death for men and women in the United States. In 2021, CVD was responsible for one in every four deaths for men and one in every five deaths for women, per the National Center for Health Statistics on the CDC WONDER database (last reviewed on April 26, 2024) [3]. While men and women have similar underlying factors for CVD, some of these factors may have stronger influences on the development of CVD in one sex versus the other [3-6].

Cardiovascular disease is a broad term for conditions that affect the cardiac muscle or the vascular system supplying the heart, brain, kidneys, and other vital organs [7]. There are many different types of CVDs, the most predominant being coronary artery disease (e.g., stable ischemic heart disease, acute coronary syndrome), ischemic stroke, peripheral arterial disease, and venous thromboembolism. Common risk factors include hypertension, hypercholesterolemia, type 2 diabetes, obesity, tobacco use, physical inactivity, family history, and diet [8]. Additionally, there are risks explicitly found in women, such as early menarche (before age 11), early menopause (before age 40), polycystic ovary syndrome, gestational diabetes, preterm delivery, deliveries of low birth weight or high birth weight in infants, hypertensive disorders of pregnancy (preeclampsia/eclampsia) and hormonal therapy [3-6]. These risk factors, particularly hypertension, hypercholesterolemia, and cigarette smoking, increase the susceptibility of atherosclerosis which is the leading cause of CVD. The pathogenesis underlying atherosclerosis is a well-studied mechanism primarily driven by oxidative stress and inflammatory lesions placed within arterial walls [9]. These phenomena, paired with endothelial dysfunction, form fatty streaks and eventually develop the characteristic atherosclerotic plaque [10]. Given that this process matures over several years, symptoms may not present until middle age, when the disease is typically advanced [11]. Common clinical findings in patients with CVD include but are not limited to obesity, angina, decreased exercise tolerance, and syncope or presyncope.

Venous thromboembolism

Venous thromboembolism (VTE), a general term used to describe both deep vein thrombosis (DVT) and pulmonary embolism (PE), is a condition that arises when a blood clot forms in a vein due to a disruption in hemostasis. It is a multifactorial chronic disease resulting from the interaction between acquired and hereditary risk factors, as described in Table 1 [12,13]. Acquired risk factors for VTE include modifiable factors like obesity, pregnancy, hormonal therapy, and immobilization, and non-modifiable factors such as advanced age. Conditions like cancer, smoking, surgery, trauma, prolonged immobilization, chronic obstructive pulmonary disease (COPD), inflammatory bowel disease, and autoimmune diseases can also trigger VTE [13-15].

**Table 1:** Acquired versus hereditary risk factors Adopted from Frischmuth: Obesity-related venous thromboembolism [13]

Acquired risk factors	Hereditary risk factors
Old age	Deficiency of antithrombin
Obesity	Deficiency of protein C
Oral contraceptives and hormone replacement therapy	Deficiency of protein S
Pregnancy and post-partum	Factor V Leiden
Cancer	Prothrombin G2021A
Surgery	
Trauma	
Infection	
Immobilization	
Acute stroke	
Acute myocardial infarction	
Chronic inflammatory diseases	

**Figure 1:**
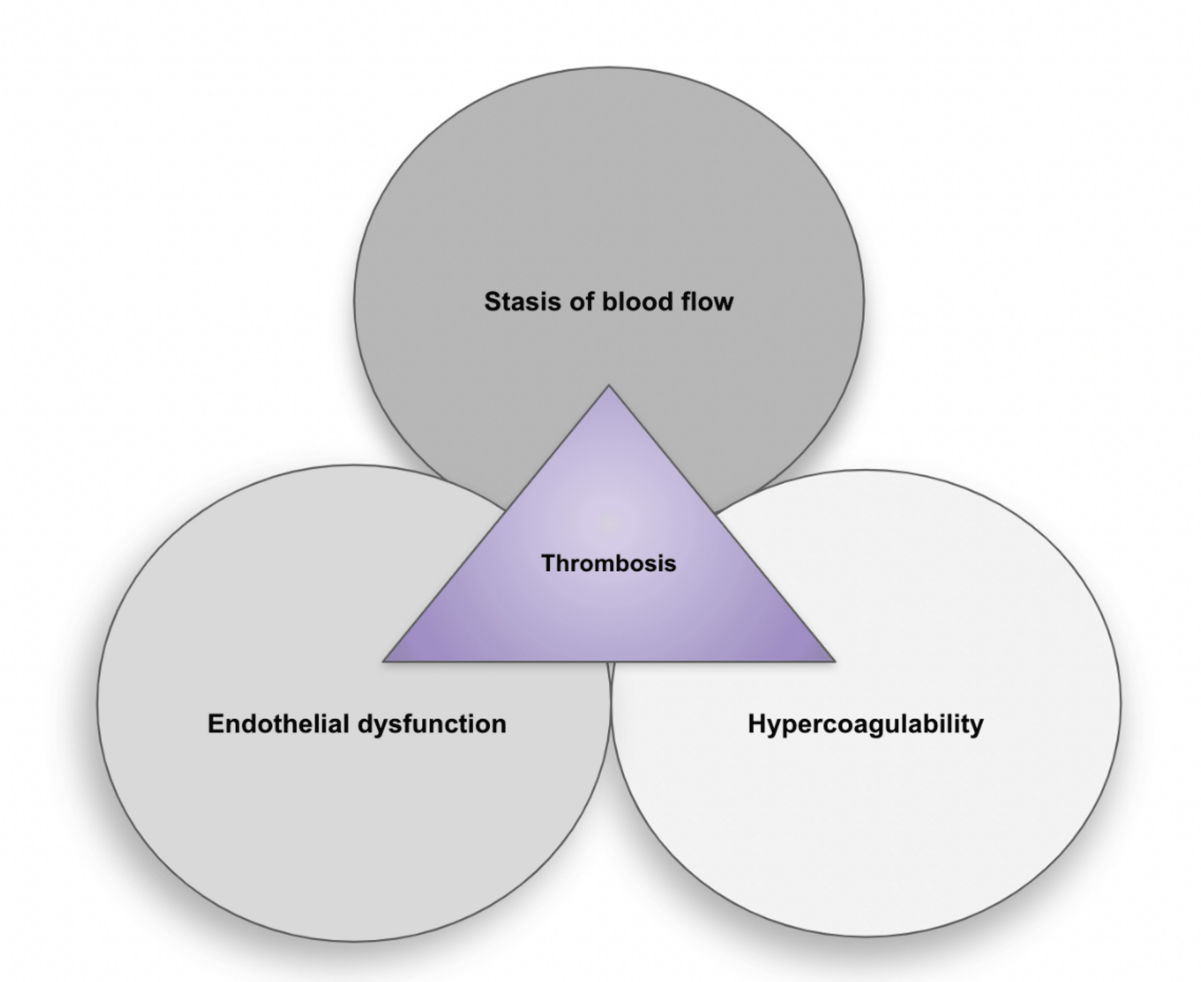
Virchow's Triad Image credit: Katherine Miller

DVT is the formation of a blood clot in the deep veins, most commonly in the lower extremities. However, this does not exclude the findings of less common origins for DVT, such as in the upper extremities and cerebral veins [16]. The prevalence of DVT in the lower limb varies by specific vein location: distal veins (40%), popliteal veins (16%), femoral veins (20%), common femoral veins (20%), and iliac veins (4%) [17]. As an entity within VTE disorders, DVT represents the third most common cause of death from cardiovascular disease after myocardial infarctions and stroke [18]. Recognizing and diagnosing DVT promptly is critical to ensure adequate treatment is administered and also for the prevention of embolization to distal parts. The pathophysiology of DVT is historically defined by the Virchow triad, which aims to delineate the contributing factors for developing venous thrombosis. The Virchow triad (Figure 1) postulates how DVT develops due to the following three mechanisms: vessel wall damage, stasis in blood flow, and a hypercoagulable state [19]. Virchow theorized that the presence of these critical factors in combination or alone increases the risk for clot development.

## Review

Clinical presentation

DVT typically presents with significant discoloration (redness or pallor), pain or tenderness, and swelling or edema in the affected limb [14,16]. However, the clinical presentation of DVT can vary greatly depending on the anatomical location of the clot, the severity of the occlusion, and the extent of thrombus involvement, as DVTs tend to grow and extend in the direction of venous blood flow [18]. Additional signs may include increased temperature of the affected limb and prominent superficial veins [20]. A hallmark finding on the physical exam includes a positive Homan's sign, expressed when passive dorsiflexion of the patient's foot elicits pain in the upper calf. However, most cases are "silent", with 50% of patients presenting as asymptomatic or with little signs or symptoms [18].

Diagnostic methods

While clinical suspicion may be high for DVT based on presenting signs and symptoms alone, other diagnostic measures must be taken into consideration. In practice, clinicians most commonly approach diagnosing DVT by referencing the Wells' criteria (Table 2). These criteria provide clinical prediction rules based on a scoring system that calculates risk factors and physical findings in patients suspected of having DVT. The generated score assists with predicting the pretest probability of DVT by categorizing patients into a high, moderate, or low probability. A score of 3 or higher indicates a high probability of DVT. Evidence supports the use of clinical algorithms to establish the pretest probability of DVT in a patient before implementing more definitive testing, which can result in potentially harmful procedures (e.g., radiation exposure or the risk of contrast-induced nephropathy) as well as increased healthcare costs [16,21]. 

**Table 2:** Clinical prediction rules to establish deep vein thrombosis (Wells' Criteria) DVT: deep vein thrombosis Note: A score of 3 or higher indicates high likelihood of DVT; a score of 1 or 2 moderate probability; a score of 0 or lower indicates a low probability Adopted from The Annals of Family Medicine Vol 5. [21]

Clinical history/finding	Score
Active cancer	1
Paralysis, paresis, or recent plaster immobilization of lower extremity	1
Recent bedridden for > 3 days or major surgery within 4 weeks	1
Localized tenderness along distribution of deep venous system	1
Entire leg swollen	1
Calf swelling greater than 3 cm compared to asymptomatic leg	1
Pitting edema (greater in symptomatic leg)	1
Collateral superficial veins (non-varicose)	1
Alternative diagnosis as likely or more possible than that of DVT	-2

Clinical prediction rules cannot exclude the diagnosis of DVT alone. Therefore, incorporating a plasma D-dimer assay into the workup for DVT also demonstrates a considerable role. D-dimer is the direct degradation product of a cross-linked fibrin clot. Thus, D-dimer levels are elevated (normal reference level is < 250 ng/mL) in conditions where endogenous fibrinolysis is activated [22]. The measured levels of D-dimer are also elevated in patients with acute VTE. They are a sensitive but nonspecific laboratory marker for DVT since elevated levels can be in different pathologic states, including malignancy, inflammatory conditions, trauma, post-operative periods, pregnancy, and liver disease [15,23]. Therefore, due to its high sensitivity, a D-dimer assay may be helpful as an exclusionary method but not for positively diagnosing DVT. In conjunction with a low pretest probability, a negative D-dimer result has a high negative predictive value for proximal DVT and precludes the need for further diagnostic imaging, which may be costly and invasive [21-23]. 

Diagnostic imaging is often used to confirm the presence of DVT further. Noninvasive diagnostic imaging strategies include compression duplex ultrasonography (US), which has proven to be the most accurate noninvasive diagnostic test and standard of care with optimal specificity [24]. It requires the use of a trained operator by radiology or vascular laboratories. Point-of-care ultrasound (POCUS) performed and interpreted by the clinician at the bedside has also become more prevalent in practice for evaluating the proximal lower extremity venous system [25]. The advantage of POCUS lies in its widespread accessibility in the emergency department, intensive care units, and medical wards. Both methods provide accurate visualization of suspected DVT without compromising the patient's comfort. US is the first-line imaging strategy because it is safe, reliable, easily accessible, and cost-effective. US can demonstrate a thrombus' size, chronicity, and degree of occlusion by using a probe to compress the suspected vein with thrombosis. If the vein of interest appears incompressible, the test is considered confirmatory. In addition, the thrombus can further be distinguished with real-time imaging using duplex or color-flow Doppler. However, the main limitation of US is its lack of ability to detect a distal DVT [15]. 

**Figure 2:**
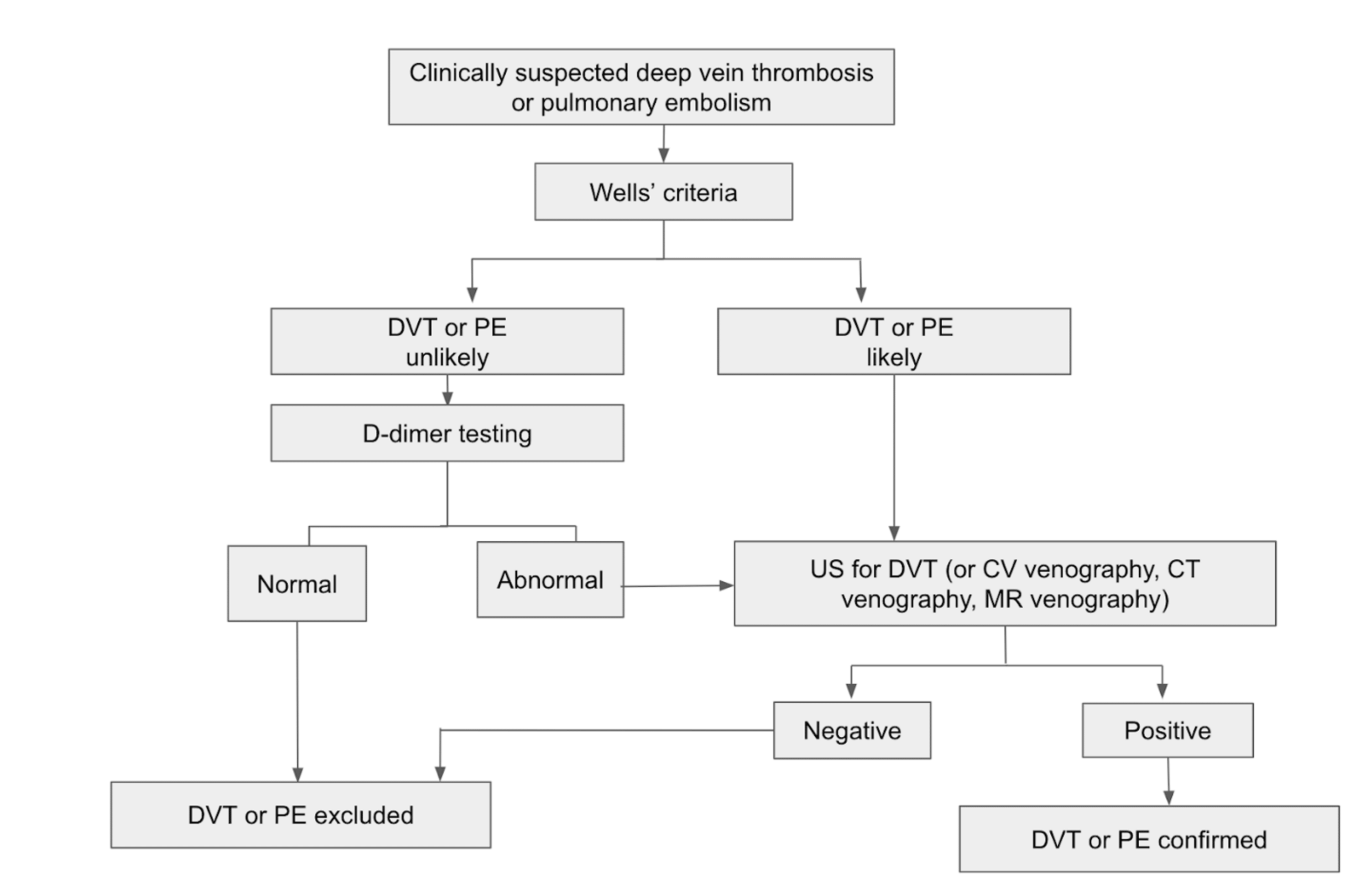
Diagnostic algorithm for suspected DVT [16] DVT: deep vein thrombosis; PE: pulmonary embolism, US: ultrasonography, CV: contrast venography, CT: computed tomography, MR: magnetic resonance Image credit: Cynthia Sanchez, Katherine Miller

Other diagnostic modalities used are contrast venography (CV), computed tomography (CT) venography, and magnetic resonance (MR) venography. CV remains the gold standard in diagnosis for DVT despite its invasive nature and association with contrast-associated risk for complications. Some of these complications include but are not limited to availability, user dependence, patient discomfort, complex visualization, and patient-specific limitations such as contrast allergy or renal insufficiency [15,16,20,21,25]. While it may be a painful procedure, it yields the highest sensitivity and specificity for suspected DVT. The CV exam is performed by inserting a cannula into a vein and injecting contrast media while taking radiographs in multiple views to visualize the deep venous system. The test is considered diagnostic if filling defects or a blockage are encountered. In CT venography, contrast media is also injected into the venous system. However, CT imaging is used to evaluate the area of interest. CV and CT venography carry the same exposure to ionizing radiation and may be limited by the use of contrast media in patients with renal insufficiency or contrast allergies. MR venography, however, does not require radiation to produce an image and is highly accurate [15,26]. This procedure uses a combination of large magnets, radio frequencies, and computers with an injection of contrast dye to produce detailed images of the venous system. While a promising alternative, its disadvantages include increased costs, operator dependability, longer scan times, and availability [15,27]. For further clarity, a diagnostic algorithm for suspected DVT is depicted below (Figure 2). 

Medical management

The main goal behind treating VTE is to improve patient symptoms, prevent the progression of DVT to PE, and eliminate thrombotic material. The standard of care for the treatment of VTE is with anticoagulant medications; however, a variety of other mechanisms are available, such as systemic thrombolytic therapy, surgical thrombectomy, catheter-directed thrombolysis (CDT), pharmacomechanical catheter-directed thrombolysis (PCDT), percutaneous aspiration thrombectomy (PAT), vena cava filter placement, venous balloon dilatation, and venous stent implantation [15,28].

The mainstay of anticoagulant treatment is with vitamin K antagonists (VKA), such as warfarin, with unfractionated heparin (UFH) or low molecular weight heparin (LMWH) utilized as bridging agents. VKAs function by competitively inhibiting vitamin K epoxide reductase complex 1, an essential enzyme necessary for activating vitamin K found in the body. As a result, VKAs deplete functional vitamin K reserves and ultimately reduce the synthesis of different active clotting factors [27]. UFH or LMWH are recommended as bridging agents due to their ability to quickly mediate anticoagulation by activating antithrombin III, thereby inactivating thrombin [29]. While UFH has a shorter half-life and is fully reversible, UFH carries an eight- to 10-fold increased risk for heparin-induced thrombocytopenia (HIT) compared to LMWH. As a result, LMWH, such as enoxaparin, is often used as the agent of choice [15]. A synthetic pentasaccharide known as fondaparinux may also be used as bridging therapy. Its mechanism of action is similar to UFH and LMWH through activation of antithrombin III. However, it accelerates its inhibition of factor Xa and has no affinity for platelet factor 4, thus further reducing the incidence of HIT. However, fondaparinux's limitations are due to its long half-life and lack of reversal agents [15]. LMWH is generally maintained for five to seven days, while VKA is recommended for at least three months [30].

Alternatively, to VKAs, direct oral anticoagulants (DOAC) have become increasingly common. DOACs such as dabigatran or rivaroxaban, apixaban, and edoxaban function by inhibiting factor IIa or factor Xa, respectively [15,31]. DOACs have been shown to be just as effective as VKAs in the treatment and prophylaxis of thromboembolic events while potentially executing a safer profile. These agents are associated with less laboratory monitoring (i.e., less major bleeding), may not require bridging time, and may have fewer drug-drug interactions. However, despite the advantages, DOACs limitations include their dependence on renal and hepatic function for clearance, long half-lives, and lack of a reversal agent [15,31].

Obesity

Overweight and obesity are commonly described as a global epidemic defined by an abnormal or excessive fat accumulation in the body that may impair health [32,33]. According to the World Health Organization, worldwide obesity has nearly tripled since 1975. Approximately 39% of adults aged 18 years or older were overweight in 2016. This corresponds to more than 1.9 billion adults worldwide. Additionally, it was estimated that over 340 million children and adolescents aged between five and 19 were overweight or obese in 2016. Overweight and obesity were once considered a high-income country problem. However, data shows that both are steadily increasing among low- and middle-income countries [32]. 

The etiology of obesity has long been researched and debated. The most simplified interpretation of obesity is defined as an energy imbalance. In other words, the energy in calories consumed is not equal to the energy in calories used by the body. While this theory still holds, further research has proposed that controlling body weight and composition is interconnected and relies on the interaction between genetic, environmental, psycho-social, and economic factors [13,34,35]. Of these factors, most are modifiable and include but are not limited to the following: epigenetics, physical inactivity, excessive caloric intake, intrauterine environment, insufficient sleep, medications, medical conditions, stress, endocrine disrupting chemicals, and the approach of healthcare professionals for overweight and obesity management [35].

Diagnostic Methods

The body mass index (BMI) is commonly used to screen and classify overweight and obesity in adults and children. In adults, BMI measures body fat based on height and weight and is expressed as the body mass (in kilograms) divided by the body height squared (in meters). In children, height and weight are expressed as percentiles and are calculated by comparing the child's BMI to growth charts for children of the same age and sex (Table 3) [33]. 

**Table 3:** Classification of body weight in adults and children BMI: body mass index BMI for adults and children [33].

For adults:	
Underweight	BMI < 18.5 kg/m^2^
Healthy weight	BMI 18.5 - 24.9 kg/m^2^
Overweight	BMI 25 - 29.9 kg/m^2^
Obese	BMI ≥ 30 kg/m^2^
For children and adolescents (ages 2-18 years):	
Underweight	BMI < 5th percentile
Healthy weight	BMI 5th - 85th percentile
Overweight	BMI 85th - 95th percentile
Obesity	BMI≥ 95th percentile

Although BMI is the most commonly used anthropometric measure, it cannot discriminate between lean and fat body mass since both body masses may weigh the same amount. As such, different techniques like waist circumference (WC), defined as the measurement of body fat distribution, pose another representation of overweight and obesity. In men, an unhealthy WC is greater than 40 inches, and in women, an unhealthy WC is greater than 35 inches [33]. The indication for using WC as a reporting measurement strongly emphasizes the causal impact that body composition has on CVD, specifically centrally predominating abdominal fat, as opposed to overall obesity. This is due to the fact that abdominal body fat is directly associated with cardiometabolic disease [36,37]. Czernichow et al. studied significant discriminators in measuring mortality risk for cardiovascular disease, measures of abdominal adiposity [WC, waist-to-hip ratio (WHR), and waist-to-height ratio (W/Hr)], but not BMI, appear to be associated more closely to CVD and relate to an increased CV mortality risk [38]. These obesity measures may help guide clinicians toward improved risk stratification and predict adverse health outcomes. 

The pervasive nature of obesity continues to place significant implications on health outcomes, as well as an increase in comorbidities that continue to rise globally. For instance, the evolution of society to a more sedentary lifestyle has fueled the steady development of the obesity epidemic. Careers have shifted from outdoor, physically demanding professions to less active office jobs. The culmination of physical inactivity and high-caloric diets seen in obese individuals are associated with atherosclerosis development and are found in comorbid conditions such as metabolic syndrome, diabetes mellitus, and hypertension. These diseases prevail heavily in CVD-affected individuals and are some of the many reasons that allude to its rising incidence in recent decades [10].

The remainder of this review will be geared towards a discussion of the relationship between patients with obesity, particularly with underlying CVD, and the ensuing course in DVT cases. Specifically, the focus will be to discuss potential biological mechanisms that demonstrate a connection between obesity and venous thromboembolism. 

Discussion

In recent years, abundant studies have continuously reported a well-established relationship between obesity and DVT. In particular, many observational studies have shown that the risk of venous thrombosis increases two to three times in individuals with higher BMI levels than those with average weights [13,39,40]. Likewise, the risk appears to increase even more in individuals who are morbidly obese (BMI >40 kg/m2) [13,39]. A meta-analysis performed by Yang et al., consisting of one cohort and eight case-control studies involving a total of 8125 patients with VTE and 23,272 controls, indicated the likelihood of a first spontaneous VTE among obesity patients was more than twice that of individuals with a normal BMI (odds ratio (OR) = 2.33; 95% confidence interval (CI), 1.68 - 3.24) [41]. This report emphasizes how the role of obesity in VTE depends not only on total body fat but specifically on the distribution of body fat, attributing visceral adiposity as the primary contributor. Similarly, Hanson et al. [42] investigated the effect of obesity and other cardiovascular risk factors on the probability of developing VTE among 792 men who were followed for 26 years. Men with the highest WC levels (>100 cm) showed an increased risk of developing a venous thromboembolic event in comparison to men with lower WC levels (<100 cm). Additionally, this study found abdominal obesity to be an independent risk factor for VTE, even when cardiovascular factors such as diabetes, stroke, high blood pressure, and myocardial infarctions were considered. Another study also showed that when comparing measures of obesity such as waist circumference, waist-to-hip ratio, hip circumference, and total body fat among 57,054 men and women, the best predictors for venous thrombosis were WC in men and HC in women [26]. As a result, individuals who demonstrate increased visceral adiposity appear to be at the highest risk for venous thrombosis [26,42,43].

The Role of Gender

Gender influences the incidence of DVT, though the precise significance remains debated in the literature. Women show higher incidence rates during their reproductive years, a factor that is crucial to consider, while men exhibit increased rates in older age groups. When these reproductive factors are considered, lifetime DVT risks appear similar between genders. This suggests that while gender-specific differences in DVT incidence exist across different age groups, the overall lifetime risk may be comparable between men and women [13]. On the contrary, Hotonleau found in a three-year retrospective case-control study including a group of 752 patients diagnosed with VTE that although women of child-bearing years similarly present a higher rate of VTE compared to men, there is still an overall predominance seen in men. After considering age and recurrence rates, findings showed that an overall 1.4-fold increased risk of VTE exists in male than female patients (OR= 5.69, respective 3.96) [12]. In a sample of hospital discharge cases analyzed over 21 years of observation out of the NHDS database, the relative risk of deep venous thrombosis, comparing obese patients with non-obese patients, was more than doubled at 2.50 (95% CI= 2.49-2.51). One considerable disadvantage to interpreting these data is their failure to provide a clear standard for defining obesity in their inclusion criterion. However, obese women were documented to have a greater relative risk for DVT than obese men with RR = 2.75 (95% CI= 2.74-2.76) versus 2.02 (95% CI= 2.01-2.04) [44].

Limiting Data

Extensive research exists to provide sufficient evidence in detailing obesity as an independent risk factor for the development of VTE [45,46,12]. However, there remains limited research and understanding underlying the biological mechanisms that correlate obesity and VTE, specifically within patients with known diagnoses of CVD. However, it is essential to note that the mechanisms associated with obesity and the development of VTE appear to be different from arterial thrombosis despite having similar cardiometabolic profiles. For instance, a population-based cohort study explored how obesity-related atherosclerotic risk factors influenced the risk of venous thrombosis and myocardial infarction and found that the association between obesity measures and risk of VTE was unaffected by the adjustment for cardiovascular risk factors, unlike the results seen for myocardial infarction [47]. Further supporting this, a prospective study encompassing both men and women without VTEs found that of the 215 validated VTE events recorded, cigarette smoking, hypertension, dyslipidemia, physical inactivity, and alcohol consumption were not associated with the risk of VTE. In contrast, obesity was found to have a significant increase in the risk for VTE [40].

Similarly, other studies have shown how obesity is a necessary factor for the apparent association between metabolic syndrome, a cluster of cardiovascular risk factors, and risk of VTE. It was seen that when the other individual features of metabolic syndrome were maintained and obesity removed, the risk between the metabolic syndrome and the development of venous thrombosis was no longer clinically significant [48,49].

Due to the limited studies found in our initial aim, the scope was narrowed to a more focused analysis to include the theories of VTE development in obesity. Despite data showing no relationship between some atherosclerotic cardiovascular disease risk factors and venous thrombosis, researchers have widely supported the implication that obesity is associated with VTE development through the disequilibrium of major molecular and cellular pathways that lead to a prothrombotic state. Several research hypotheses aim to explain the possible associations responsible for this disequilibrium (Table 4). 

**Table 4:** Mechanisms explaining the associations between venous thrombosis and obesity

Mechanisms:
Venous stasis
Chronic inflammation
Adipokines
Decreased fibrinolytic activity
Increased coagulation activity

Venous Stasis

Obesity predisposes individuals to venous stasis through several obesity-related mechanisms, such as increased intra-abdominal pressure, decreased venous blood flow, and decreased physical activity. Notable hemodynamic alterations reported include increased blood volume, CO, and systemic vascular resistance (more evident in those with hypertension and insulin resistance), which alter blood flow dynamics within systemic vasculature [50]. Obesity is also generally associated with impaired mobility and decreased physical activity, further reducing hemodynamic stability. Not only does obesity predispose individuals to venous stasis, but it is also a predisposing factor to chronic venous insufficiency. The substantial weight load experienced in overweight and obese persons functionally places added weight-bearing stress on the lower extremities and vasculature, further contributing to decreased venous blood flow. El-Menyar et al. attribute the decreased venous flow to increased femoral vein pressure, accelerating inflammation and increasing the femoral vein diameter, favoring thrombosis and venous valve dysfunction [50,51]. Central obesity is also thought to be associated with increased intra-abdominal pressures (IAP), which are consequently transferred to the extremities by the femoral veins [51]. Arvidsson et al. support this theory by showing how the pressure in the ilio-femoral vein was significantly higher in obese individuals than in controls, which also correlated with elevated urinary bladder pressure, an alternate marker for IAP [52].

Chronic Inflammation

The chronic inflammatory nature underlying these complex diseases defines one of the many mechanisms that promote prothrombosis in obesity. The chronic inflammatory pathway is driven by the highly supported notion that obesity is a systemic inflammatory disorder [53,54]. The alteration in cellular homeostasis initiates the release of specific cytokines from adipocytes, known as adipokines, that, in turn, recruit macrophages to these adipose tissue sites [54,55]. The interaction between the adipocytes and the recruited macrophages works to further promote the secretion of additional inflammatory cytokines such as tumor necrosis factor-a (TNF-a), interleukin-6 (IL-6), and IL-1B into circulation that perpetuates the systemic inflammatory state [54]. Additionally, other studies have found increasing levels of C-reactive protein (CRP) in overweight and obese patients, which indicates low-grade systemic inflammation [56-58]. Consequently, releasing these inflammatory markers affects the vascular endothelium by activating pro-coagulatory signaling cascades and downregulating anticoagulant regulatory proteins [59]. The combination of these factors activating a chronic inflammatory state sustains the proinflammatory nature of obesity. 

Decreased Fibrinolytic Activity

Obesity additionally promotes prothrombosis via impaired fibrinolysis, mainly due to the effects imposed by plasminogen activator inhibitor-1 (PAI-1). PAI-1 is responsible for inhibiting tissue plasminogen activator (tPA), the key regulator in the cleavage between plasmin and plasminogen. One study investigated the relationship between elevated plasma clot lysis time (CLT) and the risk of venous thrombosis. It was reported that PAI-1 is an independent risk factor responsible for the majority of variance in CLT seen among studied individuals. Thus, PAI-1 is one of the primary mechanisms inhibiting fibrinolysis in the body and consequently promotes venous thrombosis [39,60,61]. Adipocytes have been shown to produce PAI-1, thereby indicating a positive correlation with obesity, specifically in abdominal-type body fat distribution [62,63]. According to Eriksson et al., adipose tissue secretion of PAI-1 showed an eight-fold higher secretion in obese individuals than in non-obese individuals [64]. Other studies corroborate these findings by demonstrating that significantly higher levels of PAI-1 were seen in obese women with increased waist-hip ratios than in those with a low waist-hip ratio or lean body composition [65]. Likewise, studies that focused on the effects of weight loss in obese individuals showed up to 70% reductions in PAI-1 circulating levels when individuals lost more than 10% of their initial weight [66]. As a result, these data show convincing evidence that PAI-1 is associated with obese individuals with impaired fibrinolytic activities. 

Study Limitations

Several limitations have proven to yield confounding evidence concerning identifying patients with obesity who present with suspected DVT. In general, the diagnosis of DVT may be complex in patients with obesity due to higher BMI, which raises challenges when approaching diagnostic imaging, specifically, the method in which these studies are performed. A 2011 retrospective review, which aimed to determine which BMI duplex ultrasonography does not accurately detect DVT, suggests that patients with > 40 kg/m2 BMI may require alternative imaging modalities to diagnose the presence of venous thromboembolism [67]. Their justification is that an increased BMI is associated with an increased likelihood of an indeterminate lower extremity study. 

The limitation to diagnosing suspected DVT in obese patients should be recognized universally as well as addressed within the medical field for the following additional reasons. Patients may face weight limits of medical equipment, including physical examination tables and CT scanners [68]. While advancing technology has provided clinicians with more accommodating weight limits for these devices, it is essential to remember that not all healthcare facilities are readily accessible to these resources. Smaller or rurally located facilities may require transport to an offsite imaging center, thus delaying treatment.

As previously mentioned in this review, ultrasonography is the initial preferred choice in DVT imaging studies. However, obese patients may be subject to poor visualization of potential thrombus in venous structures due to increased soft-tissue thickness overlying the anatomy [68]. Ultrasonography relies on the technician's ability to palpate body surfaces, but a thick layer of adipose tissue obscuring bony landmarks hinders accurate positioning [69]. As a result, these patients may receive improper evaluations, rendering them low-quality and unable to aid in a clinically relevant capacity. 

There are also presenting signs on physical examinations for suspected DVT, which must be taken into consideration when evaluating obese patients. Patients with obesity may have baseline chronic dyspnea, tachypnea, tachycardia, and lower extremity edema due to body habitus that does not necessarily result from an underlying venous thromboembolism [70]. These symptoms in a lean or lower BMI patient would otherwise raise concerns for a highly suspected DVT. However, considering their history means evaluating any acute changes from their baseline. This reiterates history-taking's crucial role in approaching the evaluation of DVT. 

An additional finding in obese patients that may impose a challenge to diagnosing a DVT is that D-dimer levels, the global biomarker for coagulation activation, are higher at baseline in obesity patients [71]. This may confer the laboratory test as nonspecific; therefore, the measurement of D-dimer may be unreliable for workup, leading to a compromised clinical diagnosis. Alternative diagnostic tests should be considered for clinical correlation.

Unfortunately, these disparities place obese patients at an increased risk for insufficient investigations of a disease, which heavily relies on an accurate diagnosis promptly for the prevention of life-threatening outcomes if left untreated. The discrepancy may lead to both the underdiagnosis and performance of excessive testing for DVT in obese patients. It is also possible that this inconsistency between diagnosing average weight versus obese patients has affected the standards used for DVT diagnoses seen in the literature.

Suggestions for future research in the associations of obesity and DVT in cardiovascular individuals include studies that isolate subjects strictly defined based on their medical history. Specifically, a past medical history supports those with a known documented cardiovascular disease as well as a BMI greater than 30 kg/m2. Thorough categorization to eliminate outliers such as underweight or low BMI individuals would further ensure that research findings support a more focused study.

## Conclusions

Deep vein thrombosis is one of the most common phenomena in complicated cardiovascular disease. While a plethora of determinants are associated with DVT development, obesity serves as a moderate and independent risk factor for the development of DVT. Most of the reported cases are manifested within deep veins of lower limbs in affected individuals and present with varying symptoms from asymptomatic to classically seen signs of DVT. 

While current research shows obesity and DVT may be related through mechanisms such as venous stasis, inflammation, and hypofibrinolysis, it is not fully understood how the two variables are related in affected patients who are diagnosed explicitly with underlying CVD. Limited studies have shown, however, that the association between the two is not strongly affected by the adjustment for cardiovascular risk factors. With this in mind, one can infer that traditional atherosclerotic risk factors are not significantly correlated with venous thrombosis formation, and further research is necessary in order to effectively isolate the risk of DVT in the population of overweight and obese patients.

## References

[1] World Health Organization. (2023 (2024). Non communicable diseases [Fact sheet]. http://www.who.int/news-room/fact-sheets/detail/noncommunicable-diseases.

[2] (2024). Know the difference [Fact sheet]. http://www.nhlbi.nih.gov/resourses/know-differences-cardiovascular-disease-heart-disease-coronary-heart-disease.

[3] (2024). Women and heart disease. http://www.cdc.gov/heartdisease/women.htm.

[4] Gao Z, Chen Z, Sun A, Deng X (2019). Gender differences in cardiovascular disease. Med Nov Technol Devices.

[5] Galiuto L, Locorotondo G (2015). Gender differences in cardiovascular disease. J Integr Cardiol.

[6] Centers for Disease Control and Prevention. (2024, January 9 (2024). Men and heart disease. http://www.cdc.gov/heartdisease/men.htm.

[7] Gaziano T, Reddy KS, Paccaud F, Horton S, Chaturvedi V (2006). Cardiovascular disease. Disease Control Priorities in Developing Countries, 2nd edition.

[8] NHS NHS (2024). Cardiovascular disease. https://www.nhs.uk/conditions/cardiovascular-disease/.

[9] Scott J (2004). Pathophysiology and biochemistry of cardiovascular disease. Curr Opin Genet Dev.

[10] Lopez EO, Ballard BD, Jan A (2023). Cardiovascular disease. StatPearls [Internet].

[11] Hajar R (2017). Risk factors for coronary artery disease: historical perspectives. Heart Views.

[12] Hotoleanu C (2020). Association between obesity and venous thromboembolism. Med Pharm Rep.

[13] Frischmuth T (2023). Obesity-Related Venous Thromboembolism. http://munin.uit.no/bitstream/handle/10037/28520/thesis.pdf?sequence=6.

[14] Kearon C, Ageno W, Cannegieter SC, Cosmi B, Geersing GJ, Kyrle PA (2016). Categorization of patients as having provoked or unprovoked venous thromboembolism: guidance from the SSC of ISTH. J Thromb Haemost.

[15] Stone J, Hangge P, Albadawi H, Wallace A, Shamoun F, Knuttien MG, Oklu R (2017). Deep vein thrombosis: pathogenesis, diagnosis, and medical management. Cardiovascular Diagnosis and Therapy.

[16] Di Nisio M, van Es N, Büller HR (2016). Deep vein thrombosis and pulmonary embolism. Lancet.

[17] Stubbs MJ, Mouyis M, Thomas M (2018). Deep vein thrombosis. BMJ.

[18] Waheed SM, Kudaravalli P, Hotwagner DT (2023). Deep vein thrombosis (DVT). StatPearls [Internet].

[19] Kushner A, West WP, Suheb MZ (2022). Virchow triad. StatPearls [Internet].

[20] Kahn SR (1998). The clinical diagnosis of deep venous thrombosis: integrating incidence, risk factors, and symptoms and signs. Arch Intern Med.

[21] Segal JB, Eng J, Tamariz LJ, Bass EB (2007). Review of the evidence on diagnosis of deep venous thrombosis and pulmonary embolism. Ann Fam Med.

[22] Kelly J, Rudd A, Lewis RR, Hunt BJ (2002). Plasma D-dimers in the diagnosis of venous thromboembolism. Arch Intern Med.

[23] Scarvelis D, Wells PS (2006). Diagnosis and treatment of deep-vein thrombosis. CMAJ.

[24] Goodacre S, Sampson F, Thomas S, van Beek E, Sutton A (2005). Systematic review and meta-analysis of the diagnostic accuracy of ultrasonography for deep vein thrombosis. BMC Med Imaging.

[25] Varrias D, Palaiodimos L, Balasubramanian P (2021). The use of point-of-care ultrasound (POCUS) in the diagnosis of deep vein thrombosis. J Clin Med.

[26] Borch KH, Braekkan SK, Mathiesen EB, Njølstad I, Wilsgaard T, Størmer J, Hansen JB (2010). Anthropometric measures of obesity and risk of venous thromboembolism: the Tromso study. Arterioscler Thromb Vasc Biol.

[27] Patel S, Singh R, Preuss CV, Patel N (2022). Warfarin. StatPearls [Internet].

[28] Goktay AY, Senturk C (2017). Endovascular treatment of thrombosis and embolism. Adv Exp Med Biol.

[29] Hirsh J, Anand SS, Halperin JL, Fuster V (2001). Mechanism of action and pharmacology of unfractionated heparin. Arterioscler Thromb Vasc Biol.

[30] Agnelli G, Becattini C (2008). Treatment of DVT: how long is enough and how do you predict recurrence. J Thromb Thrombolysis.

[31] Schwarb H, Tsakiris DA (2016). New direct oral anticoagulants (DOAC) and their use today. Dent J (Basel).

[32] World Health Organization (2024). Obesity and Overweight [Fact Sheet]. https://www.who.int/news-room/fact-sheets/detail/obesity-and-overweight.

[33] (2024). What are overweight and obesity?. https://www.nhlbi.nih.gov/health/overweight-and-obesity.

[34] Martinez JA (2000). Body-weight regulation: causes of obesity. Proc Nutr Soc.

[35] Masood B, Moorthy M (2023). Causes of obesity: a review. Clin Med (Lond).

[36] Powell-Wiley TM, Poirier P, Burke LE (2021). Obesity and cardiovascular disease: a scientific statement from the American Heart Association. Circulation.

[37] Piché ME, Poirier P, Lemieux I, Després JP (2018). Overview of epidemiology and contribution of obesity and body fat distribution to cardiovascular disease: an update. Prog Cardiovasc Dis.

[38] Czernichow S, Kengne AP, Stamatakis E, Hamer M, Batty GD (2011). Body mass index, waist circumference and waist-hip ratio: which is the better discriminator of cardiovascular disease mortality risk?: evidence from an individual-participant meta-analysis of 82 864 participants from nine cohort studies. Obes Rev.

[39] Braekkan SK, Siegerink B, Lijfering WM, Hansen JB, Cannegieter SC, Rosendaal FR (2013). Role of obesity in the etiology of deep vein thrombosis and pulmonary embolism: current epidemiological insights. Semin Thromb Hemost.

[40] Tsai AW, Cushman M, Rosamond WD, Heckbert SR, Polak JF, Folsom AR (2002). Cardiovascular risk factors and venous thromboembolism incidence: the longitudinal investigation of thromboembolism etiology. Arch Intern Med.

[41] Yang G, De Staercke C, Hooper WC (2012). The effects of obesity on venous thromboembolism: a review. Open J Prev Med.

[42] Hansson PO, Eriksson H, Welin L, Svärdsudd K, Wilhelmsen L (1999). Smoking and abdominal obesity: risk factors for venous thromboembolism among middle-aged men: "the study of men born in 1913". Arch Intern Med.

[43] Ageno W, Prandoni P, Romualdi E (2006). The metabolic syndrome and the risk of venous thrombosis: a case-control study. J Thromb Haemost.

[44] Stein PD, Beemath A, Olson RE (2005). Obesity as a risk factor in venous thromboembolism. Am J Med.

[45] Wattanakit K, Lutsey PL, Bell EJ (2012). Association between cardiovascular disease risk factors and occurrence of venous thromboembolism. A time-dependent analysis. Thromb Haemost.

[46] Klovaite J, Benn M, Nordestgaard BG (2015). Obesity as a causal risk factor for deep venous thrombosis: a Mendelian randomization study. J Intern Med.

[47] Horvei LD, Brækkan SK, Mathiesen EB, Njølstad I, Wilsgaard T, Hansen JB (2014). Obesity measures and risk of venous thromboembolism and myocardial infarction. Eur J Epidemiol.

[48] Ageno W, Di Minno MN, Ay C (2014). Association between the metabolic syndrome, its individual components, and unprovoked venous thromboembolism: results of a patient-level meta-analysis. Arterioscler Thromb Vasc Biol.

[49] Ray JG, Lonn E, Yi Q (2007). Venous thromboembolism in association with features of the metabolic syndrome. QJM.

[50] Lopez-Jimenez F, Almahmeed W, Bays H (2022). Obesity and cardiovascular disease: mechanistic insights and management strategies. A joint position paper by the World Heart Federation and World Obesity Federation. Eur J Prev Cardiol.

[51] Willenberg T, Schumacher A, Amann-Vesti B (2010). Impact of obesity on venous hemodynamics of the lower limbs. J Vasc Surg.

[52] Arfvidsson B, Eklof B, Balfour J (2005). Iliofemoral venous pressure correlates with intraabdominal pressure in morbidly obese patients. Vasc Endovascular Surg.

[53] Vandanmagsar B, Youm YH, Ravussin A (2011). The NLRP3 inflammasome instigates obesity-induced inflammation and insulin resistance. Nat Med.

[54] Tchernof A, Després JP (2013). Pathophysiology of human visceral obesity: an update. Physiol Rev.

[55] Blokhin IO, Lentz SR (2013). Mechanisms of thrombosis in obesity. Curr Opin Hematol.

[56] Eichinger S, Hron G, Bialonczyk C (2008). Overweight, obesity, and the risk of recurrent venous thromboembolism. Arch Intern Med.

[57] Olson NC, Cushman M, Lutsey PL (2014). Inflammation markers and incident venous thromboembolism: the REasons for Geographic And Racial Differences in Stroke (REGARDS) cohort. J Thromb Haemost.

[58] Lindstrom S, Germain M, Crous-Bou M, Smith EN, Morange PE, van Hylckama Vlieg A, de Haan HG (2018). Correction to: Assessing the causal relationship between obesity and venous thromboembolism through a Mendelian randomization study. Hum Genet.

[59] Levi M, van der Poll T, Schultz M (2012). Infection and inflammation as risk factors for thrombosis and atherosclerosis. Semin Thromb Hemost.

[60] Darvall KA, Sam RC, Silverman SH, Bradbury AW, Adam DJ (2007). Obesity and thrombosis. Eur J Vasc Endovasc Surg.

[61] Meltzer ME, Lisman T, de Groot PG, Meijers JC, le Cessie S, Doggen CJ, Rosendaal FR (2010). Venous thrombosis risk associated with plasma hypofibrinolysis is explained by elevated plasma levels of TAFI and PAI-1. Blood.

[62] Van Gaal LF, Mertens IL, De Block CE (2006). Mechanisms linking obesity with cardiovascular disease. Nature.

[63] Skurk T, Hauner H (2004). Obesity and impaired fibrinolysis: role of adipose production of plasminogen activator inhibitor-1. Int J Obes Relat Metab Disord.

[64] Eriksson P, Reynisdottir S, Lönnqvist F, Stemme V, Hamsten A, Arner P (1998). Adipose tissue secretion of plasminogen activator inhibitor-1 in non-obese and obese individuals. Diabetologia.

[65] Landin K, Stigendal L, Eriksson E, Krotkiewski M, Risberg B, Tengborn L, Smith U (1990). Abdominal obesity is associated with an impaired fibrinolytic activity and elevated plasminogen activator inhibitor-1. Metabolism.

[66] Mertens I, Van Gaal LF (2002). Obesity, haemostasis and the fibrinolytic system. Obes Rev.

[67] Dua A, Desai SS, Nodel A, Heller JA (2015). The impact of body mass index on lower extremity duplex ultrasonography for deep vein thrombosis diagnosis. Ann Vasc Surg.

[68] Cascio V, Hon M, Haramati LB, Gour A, Spiegler P, Bhalla S, Katz DS (2018). Imaging of suspected pulmonary embolism and deep venous thrombosis in obese patients. Br J Radiol.

[69] Le NT, Robinson J, Lewis SJ (2015). Obese patients and radiography literature: what do we know about a big issue?. J Med Radiat Sci.

[70] Bindlish S, Ng J, Ghusn W, Fitch A, Bays HE (2023). Obesity, thrombosis, venous disease, lymphatic disease, and lipedema: an Obesity Medicine Association (OMA) clinical practice statement (CPS) 2023. Obes Pillars.

[71] Hansen ES, Rinde FB, Edvardsen MS (2021). Elevated plasma D-dimer levels are associated with risk of future incident venous thromboembolism. Thromb Res.

